# The Different Molecular Code in Generation of Dopaminergic Neurons from Astrocytes and Mesenchymal Stem Cells

**DOI:** 10.3390/ijms222212141

**Published:** 2021-11-09

**Authors:** Nana Wang, Xingrui Ji, Yue Wu, Shaocong Zhou, Huiyu Peng, Jingwen Wang, Shuang Yu, Jingzhong Zhang

**Affiliations:** 1 School of Biomedical Engineering (Suzhou), Division of Life Sciences and Medicine, University of Science and Technology of China, Hefei 230026, China; wangnana93@mail.ustc.edu.cn (N.W.); jxr2019@mail.ustc.edu.cn (X.J.); zhousc97@mail.ustc.edu.cn (S.Z.); 15005215936@163.com (H.P.); 2 Suzhou Institute of Biomedical Engineering and Technology, Chinese Academy of Sciences, Suzhou 215163, China; wuy@sibet.ac.cn (Y.W.); wangjw@sibet.ac.cn (J.W.); 3 Zhengzhou Zhongke Institute of Biomedical Engineering and Technology, Zhengzhou 450001, China

**Keywords:** PD, AS, MSCs, DA candidate, transcription factors, small molecules, morphogens

## Abstract

Transplantation of exogenous dopaminergic (DA) neurons is an alternative strategy to replenish DA neurons that have lost along the course of Parkinson’s disease (PD). From the perspective of ethical acceptation, the source limitations, and the intrinsic features of PD pathology, astrocytes (AS) and mesenchymal stem cells (MSCs) are the two promising candidates of DA induction. In the present study, we induced AS or MSCs primary culture by the combination of the classical transcription-factor cocktails Mash1, Lmx1a, and Nurr1 (MLN), the chemical cocktails (S/C/D), and the morphogens SHH, FGF8, and FGF2 (S/F8/F2); the efficiency of induction into DA neurons was further analyzed by using immunostaining against the DA neuronal markers. AS could be efficiently converted into the DA neurons in vitro by the transcriptional regulation of MLN, and the combination with S/C/D or S/F8/F2 further increased the conversion efficiency. In contrast, MSCs from umbilical cord (UC-MSCs) or adipose tissue (AD-MSCs) showed moderate TH immunoreactivity after the induction with S/F8/F2 instead of with MLN or S/C/D. Our data demonstrated that AS and MSCs held lineage-specific molecular codes on the induction into DA neurons and highlighted the unique superiority of AS in the potential of cell replacement therapy for PD.

## 1. Introduction

Parkinson’s disease (PD) is the second most prevalent neurodegenerative disorder, and the core pathology is characterized by the selective degeneration of dopaminergic (DA) neurons in the midbrain [1,2]. Over the last few decades, the mainstay of clinical therapy focused on dopaminergic agents, which facilitated the dopamine restoration and rejuvenation of motor functions. However, these medications failed to promote the recovery of the lost DA neurons; meanwhile, some undesirable side effects in the later stage of treatment often occur, such as dyskinesia [3]. Replenishment of the dopamine-producing cells is considered to be a plausible strategy that improves focal dopamine levels, accompanied by the restoration of the nigrostriatal function, and the prerequisite for the DA neurons candidate is the easy access, safety as well as the capacity of inducting into DA neurons.

The early studies were performed using fetal ventral mesencephalic tissue for grafting [4,5], but the ethical concerns and availability impede the clinical application. Although some evidence has revealed that embryonic stem cells (ESCs)-derived DA neurons could ameliorate dyskinesia in the animal models of PD [6], there is much dispute about the feasibility of such a strategy due to the safety issues. Similar limitations also exist in cell therapy with neural stem cells [7] and pluripotent induced stem cells (iPSCs) [3,8,9]. In contrast, mesenchymal stem cells (MSCs) isolated from the umbilical cord (UC-MSCs), adipose tissue (AD-MSCs), bone marrow (BM-MSCs), and other tissue sources [10] have displayed considerable superiorities of lower immunogenicity, less tumorigenicity, and higher ethical acceptance [5,11]. A series of studies have been reported that MSCs were able to rescue nigrostriatal circuits and improve behavioral performance in the PD models [12], which were considered as one of the ideal donor cells for PD treatment [13].

The key to intracerebral transplantation is whether the engrafted cells can establish functional integration with the host brain. Note that astrocytes (AS) are to bear the brunt of the responsibility in the process [14]. It has been suggested that progressive degeneration of dopamine neurons partly resulted from persistent AS activation [15]. The recent discoveries have confirmed that DA neurons can be directly induced from various cell types by genetic manipulation [16,17], and AS has attracted special attention due to the distribution and critical role in the regulation of neuronal function [18,19,20]. The strategy for direct cell types conversion opens a new avenue in PD, suggesting the feasibility of the high yields of functional DA neurons from AS for cell transplantation.

Over the last few decades, a variety of in vitro induction paradigm was established for the induction into DA neurons, including multiple molecules affecting both the intrinsic transcriptional regulation and the extrinsic microenvironment state [21,22,23,24]. For example, the morphogens such as sonic hedgehog (SHH), fibroblast growth factor 8 (FGF8), and basic fibroblast growth factor (FGF2) represent the floor plate-based strategy for the induction into DA neurons [25,26] and are essential for the generation of stem cell-derived DA neurons [24,25,27]; meanwhile, these morphogens also were supplemented in induction paradigm of cell fate conversion of somatic cells to increase the efficiency of DA neurons [19]. Furthermore, transcription factors, such as achaete-scute family bHLH transcription factor 1 (Ascl1, also known as Mash1), lim homeobox transcription factor 1 alpha (Lmx1a), nuclear receptor subfamily 4 group a member 2 (Nurr1), and neurogenin 2 (Ngn2), are key determinants in DA specification [28,29,30]. As for DA neurons, it had already been shown by many reports that fibroblasts were directly converted into DA neurons by a combination of multiple transcription factors (Mash1, paired like homeodomain 3 (Pitx3), engrailed homeobox 1 (En1), Nurr1, Lmx1a, and forkhead box a2 (Foxa2)) [31] or (Mash1, Pitx3, Nurr1, Ngn2, and sry-box transcription factor 2 (SOX2)) [32] or (Mash1, Lmx1a, and Nurr1 (collectively known as “MLN”)) [16]. Especially, the transcriptional regulation by “MLN” factors was the most extensively applied combination for DA induction in various cell types [16,19,33]. Notably, more and more proofs have suggested that small molecules could facilitate the derivation from MSCs or somatic cells, such as SB431542, which can inhibit the transforming growth factor β (TGFβ) signaling from inducing expression of downstream TGFβ targets following promoting the efficient neuronal conversion of non-neurotic cells [34,35,36]. CHIR99021, as a potent glycogen synthase kinase 3β (GSK-3β) inhibitor, can strongly activate the WNT signal pathway [37], canonical WNT signaling plays a key role in regional specification [25]. In addition, DAPT, a Notch inhibitor, was reported to be involved in the differentiation program, organ formation, and morphogenesis [38]. Previously, the Gong Chen lab has successfully demonstrated that the combination of SB431542, CHIR99021, and DAPT (S/C/D) could convert AS into neurons [34]. Therefore, the three classical combinations (SHH, FGF8, and FGF2, S/F8/F2; MLN; S/C/D) were applied to measure the capacity of AS and MSCs induction into DA neurons.

Given the fact that there are few studies that put forward a direct comparison of DA-producing capacities between MSCs and AS, the two promising candidates in the cell therapy for PD, in the present study, we compared the efficiency of MSCs and AS inducting into DA neurons, respectively, by the classical transcription factor-based regulation (MLN) [16], chemical modulation (S/C/D) [34], and morphogen-based modulation (S/F8/F2) [24,25,39]. Unexpectedly, AS showed amazing efficiency of converting into DA neurons with mature phenotype by the MLN transcriptional induction w/o chemicals S/C/D, while MSCs were only induced into immature neurons with unspecialized DA phenotypes. An optimized induction with morphogens S/F8/F2 could induct MSCs into TH-expressing (tyrosine hydroxylase) cells; however, the expression level of TH in MSC-derived DA neurons was significantly lower than that in AS-converted DA neurons. These data suggested that AS and MSCs held lineage-specific molecular codes on the induction into DA neurons, and AS showed great potential in the generation of DA-producing neurons.

## 2. Results

### 2.1. Characterization of AS and MSCs Primary Culture

**Figure 1:**
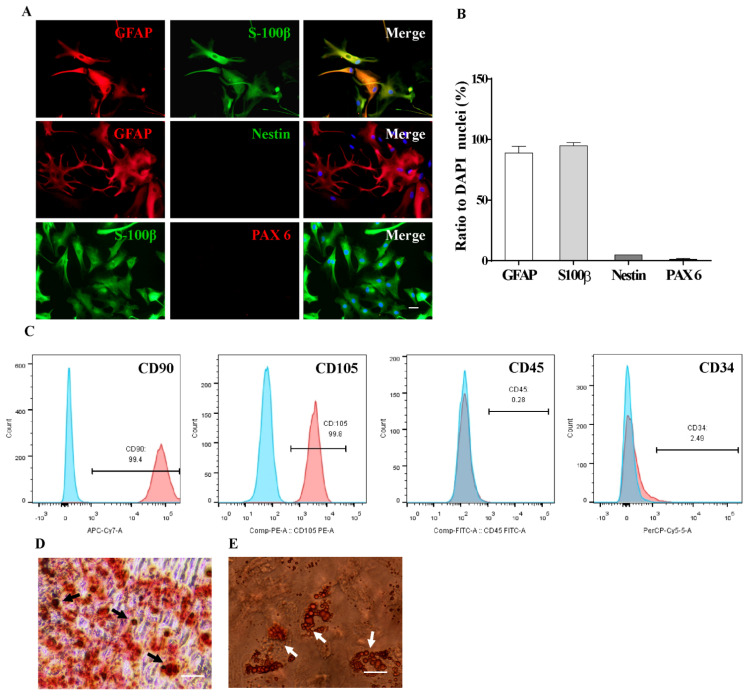
Characterization of primary cultured AS and MSCs. (**A**) Immunostaining analysis showed that nearly 95% of cells in the primary AS culture were immuno-positive for astrocytic markers GFAP and S-100β, only a small percentage of cells expressed makers for neural progenitor Nestin and PAX6. (**B**) Statistical analysis showed that the overwhelming majority of cells in the primary AS culture expressed astrocytic markers (*n* = 3 independent experiments). (**C**) MSCs were stained with surface markers CD90, CD105, CD45, and CD34 and subjected to flow cytometry analysis. More than 99% of cells are immunoreactive to mesenchymal stem cell markers CD90 and CD105 and negative for hematopoietic markers CD45 and CD34. (**D**) The osteogenic differentiation of MSCs was evaluated by Alizarin red staining. Note that the black arrows indicate the calcified tubercles. (**E**) The lipogenic differentiation of MSCs was detected by oil red staining, and the white arrows indicate lipid droplets. Scale bars: 20 µm in (**A**), 50 µm in (**C**,**D**). Numerical data represent mean ± SEM.

To minimize the possible influence of neural progenitors on the subsequent experiments [40], the primary astrocytic culture from rat hippocampus was purified [41] and maintained in AS growth medium containing 10% fetal bovine serum (FBS) and 5 ng/mL FGF2. As shown in Figure 1A,B, nearly 95% of cells expressed astrocytic markers, glial fibrillary acidic protein (GFAP), and s100 calcium binding protein β (S100β), only less than 5% of cells were positive for the markers of neural progenitors, i.e., Nestin and paired box-6 (PAX6). These results showed that the harvested cells were purified AS indeed.

Next, according to the minimum standards of human MSCs characterization [42], the phenotypes of MSCs were evaluated by flow cytometry. More than 99% of MSCs were immunoreactive to the mesenchymal markers CD90 and CD105, and less than 3% of cells express hematopoietic stem cell markers CD45 and CD34 (Figure 1C). As the multipotent progenitor cells, MSCs are capable of differentiating into osteoblastic or adipocytic lineages under certain conditions, and the differentiation capacity was evaluated by oil red O or alizarin red staining, respectively (Figure 1D,E). Note that lipid droplets or calcified nodules could be observed in the differentiated MSCs. These results indicated that primary MSCs fulfilled the criteria of MSCs.

### 2.2. AS but Not MSCs Were Efficiently Inducted into DA Neurons by Transcriptional Regulation

**Figure 2:**
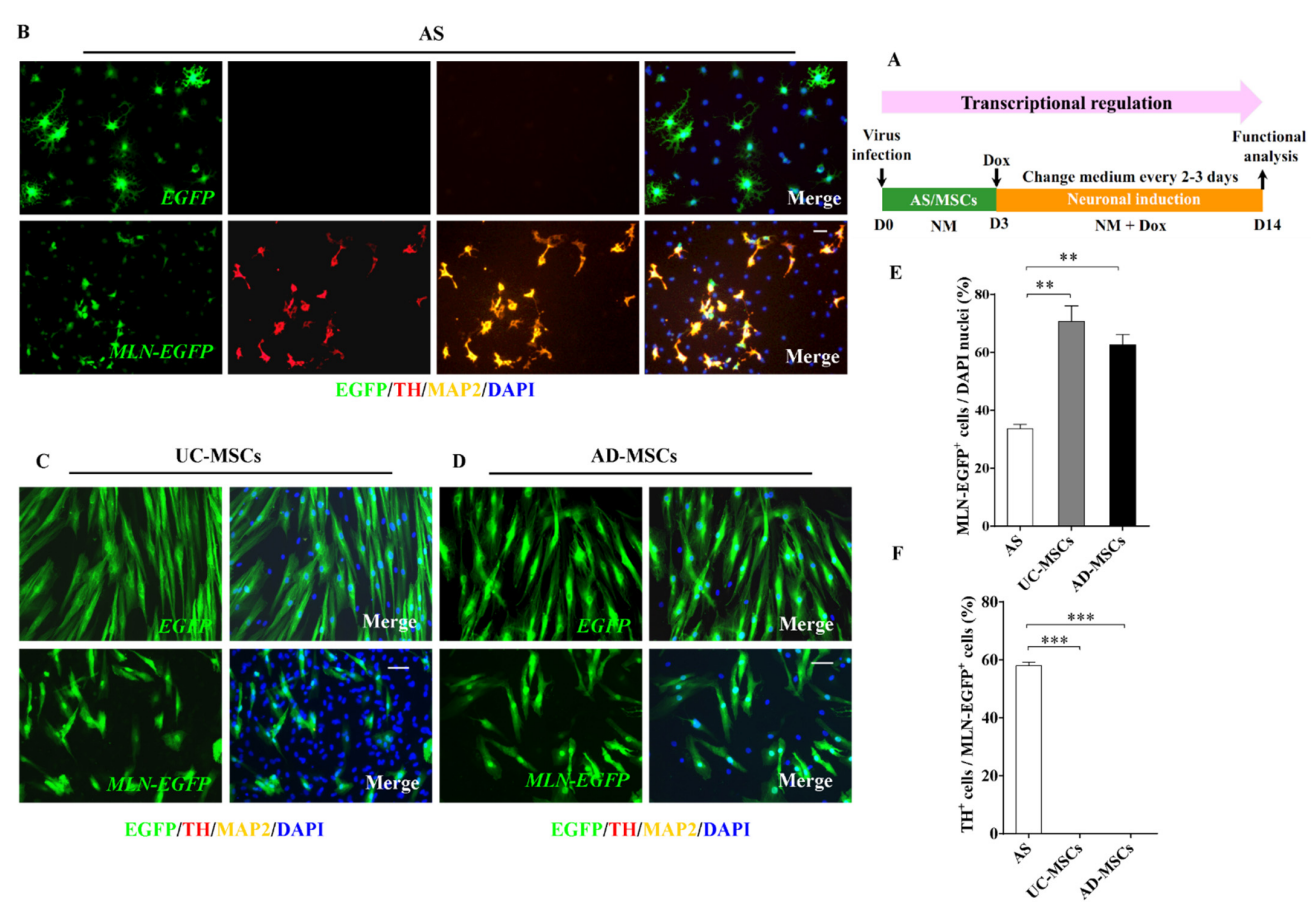
Induction of AS but not MSCs into DA neurons through a classical three-transcription-factor cocktail (Mash1, Lmx1a, Nurr1, collectively known as “MLN”). (**A**) The paradigm of the transcription factor-based induction of AS or MSCs into DA neurons. AS, UC-MSCs, or AD-MSCs were first transferred into the neuronal induction medium (NM) and infected with lentivirus carrying the reverse Tet-transactivator (*FUW-rtTA2*) and *Tet-O-FUW-Mash1-P2A-Lmx1a-F2A-Nurr1-IRES-EGFP* (*MLN-EGFP*). Dox was administered from the 3rd day (D3) to initiate the transcription of the MLN cocktail. Cells infected with lentivirus carrying *FUW-rtTA2* and *Tet-O-IRES-EGFP* (*EGFP*) were used as a control. A total of 14 days later (D14), the induced AS, UC-MSCs, and AD-MSCs were stained with EGFP, TH, and MAP2 antibodies, and the representative images were shown in panel (**B**) (AS), (**C**) (UC-MSCs), and (**D**) (AD-MSCs). DAPI (blue) was used as counterstaining. EGFP-positive cells could be observed in all the *MLN-EGFP* or *EGFP*-infected cells, including AS, UC-MSCs, and AD-MSCs (**B**–**D**); however, TH- and MAP2-positive DA neurons were detected only in the transcription factor-based induction system for AS (**B**), but not in the one for UC-MSCs (**C**) or AD-MSCs (**D**). (**E**) Numerical analysis revealed that the percentage of MLN-EGFP-positive cells in the AS culture is significantly lower than those in UC-MSCs or AD-MSCs cultures (*n* = 3 independent experiments). (**F**) Quantitative analysis revealed significantly more TH-positive neurons were induced from MLN-EGFP-positive AS on D14, as compared to those observed in UC-MSCs and AD-MSCs groups (*n* = 3 independent experiments). Scale bars: 50 µm in (**B**–**D**). mean ± SEM. ** indicates *p* < 0.01, *** indicates *p* < 0.001 in AS culture vs. UC-MSCs or AD-MSCs.

Transcription factors overexpression is the dominant way to accomplish the cell fate conversion [43]. In the present study, AS and MSCs were infected, respectively, with lentivirus carrying the reverse Tet-transactivator (*FUW-rtTA2*) and *MLN-EGFP*, followed by the activation of exogenous genes with doxycycline (Dox) (Figure 2A). The lentivirus carrying *FUW-rtTA2* and *EGFP* was used as a control.

A total of 7 days after infection, simple neuronal-like cells started to be observed in the *MLN-EGFP*-infected AS. None of such morphology change was observed in the *MLN-EGFP*-infected MSCs or *EGFP*-infected AS/MSCs (Figure 2B–D). A total of 14 days after the infection, triple staining against EGFP, TH, and MAP2 (microtubule associated protein 2) showed that many of the *MLN-EGFP*-infected AS expressed both TH, the specific marker of DA neurons, and MAP2, the marker of mature neurons (Figure 2B). Neither TH nor MAP2 was detected in the *EGFP*-infected AS (Figure 2B). In contrast, although MSCs possess multi-differentiation capacity, none of TH or MAP2 phenotypes could be observed in *MLN-EGFP* or *EGFP*-infected UC-MSCs (Figure 2C) or AD-MSCs (Figure 2D).

To avoid the influence of infection efficiency on the induction rates of AS or MSCs, the percentages of the *MLN-EGFP*-infected cells were compared among AS, UC-MSCs, and AD-MSCs cultures. As shown in Figure 2E, the infection rate in AS culture was actually the lowest (Figure 2E, *p* < 0.01). Statistical analysis further revealed that TH-positive neurons could be converted in about 60% of the *MLN-EGFP*-infected AS (Figure 2F, *p* < 0.001), indicating that the established induction system with transcriptional factors efficiently converted AS into DA neurons, however, neither AD-MSCs nor UC-MSCs showed any response toward DA induction under MLN transcriptional regulation.

### 2.3. Higher Neuronal Plasticity of AS Compared with MSCs by Chemical Modulation

**Figure 3:**
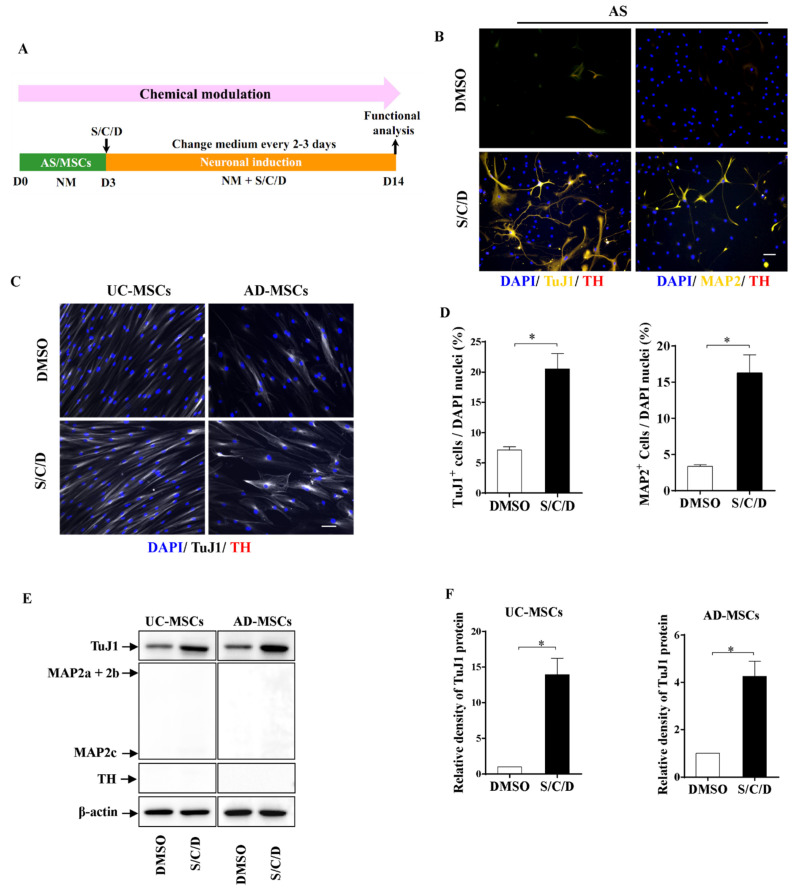
AS and MSCs acquired neuronal phenotypes under chemical modulation. (**A**) The paradigm of chemical modulation. AS, UC-MSCs, or AD-MSCs was primed in NM for 3 days, followed by induction with chemicals SB431542, CHIR99021, and DAPT (S/C/D). On the 14th day, cells were fixed and analyzed by immunostaining or WB. (**B**) Representative immunostaining images of DMSO-treated (upper panel) and S/C/D-treated AS (lower panel). There are many cells expressing TuJ1 or MAP2 in the S/C/D-treated AS, markers of the immature and mature neurons, respectively. In contrast, few cells expressed these two markers in the DMSO-treated AS. None of the TH-positive DA neurons could be observed in the DMSO- or S/C/D-induced AS culture. (**C**) Representative images of DMSO-treated (upper panel) and S/C/D-treated MSCs (lower panel) subjected to TuJ1 and TH double immunostaining. Both UC-MSCs (left column) and AD-MSCs (right column) showed no obvious changes in cell morphology, albeit increasing TuJ1 expression pattern after S/C/D treatment. None of the TH^+^ cells could be detected in the MSCs. (**D**) Quantification analysis revealed that S/C/D treatment significantly increased the percentages of TuJ1-positive cells and MAP2-positive cells in the AS culture, as compared to the DMSO-treated group (*n* = 3 independent experiments). (**E**) Immunoblotting assay for TuJ1 (50 kDa), MAP2 (high-molecular-weight MAP2 at 280 kDa, low-molecular-weight MAP2 at 70 kDa), and TH (60 kDa) in DMSO- and S/C/D-treated UC-MSCs and AD-MSCs after 14-day induction. Note that neither MAP2- nor TH-positive bands could be observed in these MSCs treated with DMSO or S/C/D. (**F**) Numerical analysis showed that the expression levels of TuJ1 in MSCs were significantly increased under S/C/D treatment, as compared to the levels in DMSO-treated MSCs (*n* = 3 independent experiments). Scale bars: 50 µm in (**B**,**C**). Data represented as mean ± SEM, * *p* < 0.05 in S/C/D-treated cells vs. the DMSO-treated ones.

It has been reported that small molecules can change cell fates [40,44]. The approach is considered as chemical genetics that uses modulators of specific signaling pathways or epigenetic modification to reverse lineage commitment [44]. Therefore, we next investigated whether AS and MSCs have the potentials for induction into DA neurons by the treatment with chemicals (S/C/D). AS and MSCs were inducted as the schematic procedure shown in Figure 3A. Immunostaining of neuron-specific class III beta-tubulin (TuJ1), a marker of neurons at an early stage, MAP2, and TH (Figure 3B) showed that significantly more of AS had committed to neuronal conversion 14 days after the S/C/D treatment as compared to the control treated with DMSO solvent, including approximately 20% TuJ1-positive cells and approximately 15% MAP2-positive cells (Figure 3D, *p* < 0.05). None of the TH-positive cells could be observed in the S/C/D-treated astrocytic culture (Figure 3B). Note that the S/C/D-treated AS showed complicated neuronal morphology with more protrusions but failed to accomplish DA lineage commitment.

Different from that AS exhibited markers of mature neuron MAP2, there were no MAP2-positive cells in UC-MSCs and AD-MSCs cultures treated with S/C/D. MSCs remained very flat, symmetrical, and spindle-shaped after S/C/D induction, which did not display a significant difference with the DMSO-treated control group (Figure 3C). The Western blot analyses in Figure 3F showed that the expression of TuJ1 protein was significantly upregulated in UC-MSCs and AD-MSCs 14 days after the induction S/C/D cocktails (Figure 3F, *p* < 0.05), but the DA markers TH and MAP2 was not present in S/C/D-treated MSCs (Figure 3E). Summarily, these results indicated that the chemical modulation by S/C/D failed to convert AS or MSCs into DA neurons; meanwhile, AS possessed higher plasticity of neuronal induction as compared with MSCs under the treatment of small molecules.

### 2.4. The Different Role of Morphogens SHH, FGF8, and FGF2 in Induction MSCs and AS into Dopaminergic Neuron-like Cells

**Figure 4:**
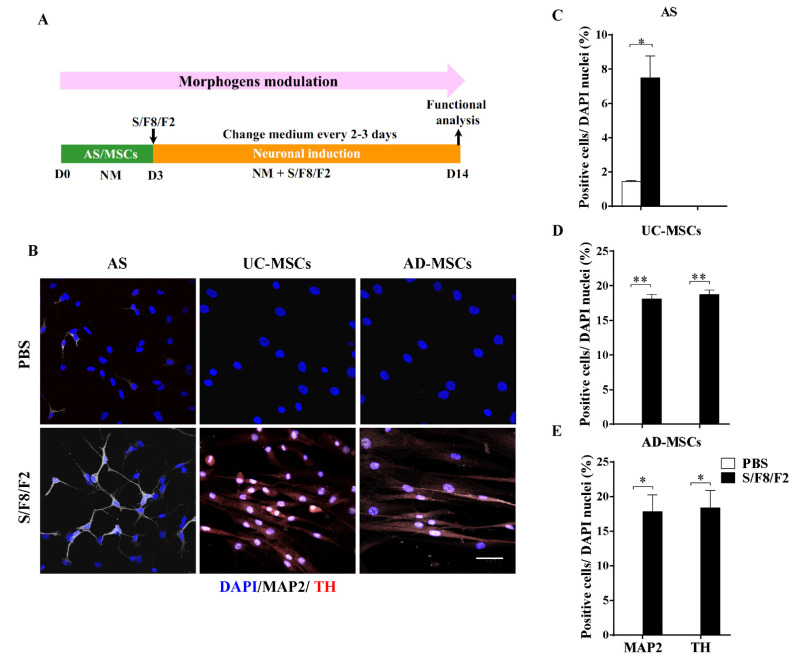
AS and MSCs showed different DA induction potential to the signaling molecules SHH, FGF8, and FGF2. (**A**) AS or MSCs were primed in NM for 3 days, followed by DA induction with signaling molecules SHH, FGF8, and FGF2 (S/F8/F2) till the 14th day. On the 14th day, cells were fixed and subjected to MAP2 and TH immunostaining. (**B**) The representative images of MAP2 and TH double staining showing AS (left column), UC-MSCs (middle column), and AD-MSCs (right column) treated with PBS (upper panel) or S/F8/F2 (lower panel) for 14 days. There were few MAP2- or TH-positive cells in all the PBS-treated cells. S/F8/F2 increased the expression of MAP2 in all three cell types, but only UC-MSCs and AD-MSCs expressed DA-specific marker TH. (**C**–**E**) The percentages of MAP2- and TH-positive cells induced from AS (**C**), UC-MSCs (**D**), AD-MSCs (**E**) were counted and compared at day 14. Positive cells are included only when the staining was distributed in the nuclei as well as cytoplasm (*n* = 3 independent experiments). Scale bars: 50 µm in (**B**). Data represented as mean ± SEM, * *p* < 0.05, ** *p* < 0.01 in S/F8/F2-treated cells vs. the corresponding PBS-treated control.

The development of DA neurons depends on not only the diverse fate-determining transcription factors and small molecules but also the morphogens, especially SHH and FGF8. Additionally, FGF2 was reported to promote neurogenesis and enhance differentiation and survival of DA neurons [45]. There, the morphogens SHH and FGF8, along with FGF2 (S/F8/F2), were administrated to AS or MSCs according to the procedure in Figure 4A, and the solvent PBS was used as a control. Double immunostaining against TH and MAP2 showed that a small portion of MAP2-positive cells with simple neurites was observed in the inducted AS, but there were no TH-expressing cells 14 days after induction (Figure 4B,C). Noticeably, under the same conditions, TH was found to be dimly positive in UC-MSCs and AD-MSCs, although these cells did not show significant morphological changes (Figure 4B). The statistical analysis revealed that MAP2-positive or TH-positive neurons could be converted to more than 15% after the addition of morphogens S/F8/F2 in either UC-MSCs (Figure 4D, *p* < 0.01) or AD-MSCs (Figure 4E, *p* < 0.05). Therefore, these results suggested that the key morphogens S/F8/F2 played differential roles in the induction of AS or MSCs into DA neuron-like cells. Note that the UC-MSCs or AD-MSCs treated with S/F8/F2 remained bipolar non-neuronal morphology although they expressed MAP2 and TH, indicating that the induced cells probably shared limited features of the functional DA neurons.

### 2.5. Enhanced Induction Efficiency and Maturation of DA Neurons by the Combination of Transcriptional and Chemical Regulation

**Figure 5:**
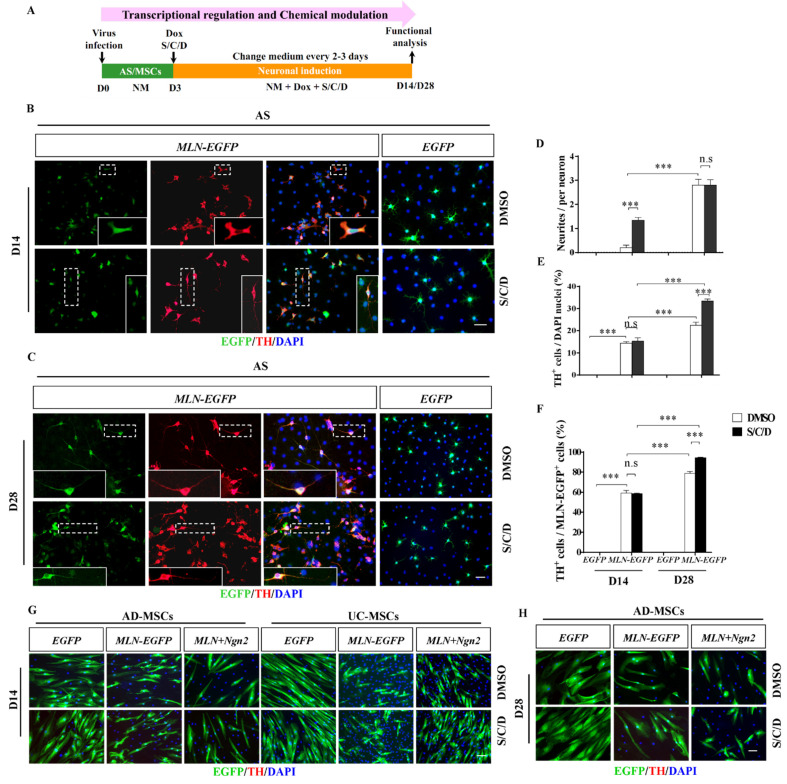
Improvement of the DA induction efficiency and maturation in AS but not MSCs by a combination of the transcriptional and the chemical modulation. (**A**) The paradigm of DA induction by the transcriptional regulation and the chemical modulation. AS, UC-MSCs, or AD-MSCs were infected with lentivirus carrying *MLN-EGFP* or *EGFP* (control) and then treated with NM containing Dox and chemicals S/C/D to initiate both the transcriptional and chemical induction. The induction system was terminated and stained for various DA markers on the 14th or 28th day for functional analysis. (**B**,**C**) The representative confocal images of AS being treated with *MLN* cocktail and S/C/D for 14 days (**B**) and 28 days (**C**). Cells were double stained for EGFP (infected cells), TH (DA neurons), and DAPI (cell nuclei). The area in the dashed box was enlarged and shown in the insets of each figure. AS infected with *EGFP* showed none of the TH-positive staining (the right column); in contrast, all the AS infected with *MLN* cocktail are stained positive for TH (the left 3 columns). Note that in Figure 5B, the induced TH-positive neurons extended more of neurites with the addition of S/C/D (the lower panel), as compared to the corresponding DMSO-treated group (the upper panel). (**D**) Numerical analysis of the neurites per TH-positive cell in the AS culture induced with *MLN* cocktail w/o S/C/D. The neurites with a length over 100 μm are included. (**E**) The effects of *MLN* cocktail and S/C/D treatment on the percentages of TH-positive cells in the AS induction system (*n* = 3 independent experiments). (**F**) The effects of *MLN* cocktail and S/C/D treatment on the percentages of TH-positive cells in the *MLN-EGFP*-infected AS (*n* = 3 independent experiments). (**G**,**H**) The representative images of AD-MSCs or UC-MSCs were infected with lentivirus carrying *EGFP*, *MLN-EGFP*, or *MLN-EGFP* with *Tet-O-Ngn2-IRES-EGFP* (*Ngn2*), followed by treatment with DMSO or S/C/D for 14 days (**G**) or 28 days (**H**). Cells were double stained for EGFP and TH. Note that EGFP fluorescence could be detected in both AD-MSCs and UC-MSCs culture, but none of TH-positive cells could be observed. Scale bars: 50 µm in (**B**,**C**,**G**,**H**). Data represented as mean ± SEM. n.s. indicates not significantly different. *** *p* < 0.001.

Given the critical roles of both transcription factors and small molecules in neuronal fate decisions, we next examined whether the induction efficiency could be further increased by transcription factors coupled with chemical cocktails in accordance with the procedure in Figure 5A. When *MLN-EGFP*-infected AS was treated with S/C/D for 14 days, double staining against TH and EGFP revealed that the induced TH-positive neurons extended long neurites, which could not be observed in *MLN-EGFP*-infected AS (Figure 4B). Although the percentages of TH-positive neurons did not differ significantly (*p* > 0.05) between the *MLN-EGFP*+DMSO- and *MLN-EGFP*+S/C/D-treated groups (Figure 5E,F), the quantification of neurites over 100 μm on each of AS-derived DA neurons revealed that the addition of S/C/D could significantly (*p* < 0.001) increase the number of neurites (>100 μm) in *MLN-EGFP* infected neurons (Figure 5D).

When the induction time was extended to 28 days (Figure 5A), more TH-positive cells with bipolar or multipolar neurites were observed (Figure 5C). More importantly, AS treated with *MLN-EGFP* and S/C/D produced significantly more (*p* < 0.001) of TH-positive neurons, as compared to the one treated with *MLN-EGFP* alone (Figure 5E,F). Note that the induction efficiency was up to approximately 90% in the *MLN-EGFP*-infected AS with the addition of S/C/D (Figure 5E,F). These observations indicated that S/C/D and *MLN* cocktail acted synergically for the DA phenotype acquisition; S/C/D promote the maturation of neurons, which in turn increased the induction of the DA phenotype.

Although MSCs have been shown to be effective for treating PD [46], the outcome of MSCs into DA lineage remained rather ambiguous. A better understanding of the mechanisms of MSCs in PD is a precondition for MSCs-based cell therapy. Here, we further examined whether MSCs were equipped with the capability of induction into DA neurons through internal and external synergy in vitro. As shown by the absence of TH, neither UC-MSCs nor AD-MSCs accomplished DA induction under the *EGFP* or *MLN-EGFP* or *MLN-EGFP* plus *Ngn2* infected coupled with S/C/D treatment (Figure 5G). Although we extended the drug treatment time and prolonged the induction time (UC-MSCs did not survive up to day 28 in the induction process), disappointingly, none of the DA neurons phenotypes were observed (Figure 5H). Collectively, the present results indicated that neither AD-MSCs nor UC-MSCs could induct into DA neurons through the dual transcriptional and chemical modulation.

### 2.6. The Role of Transcription Factors and Morphogens in the Induction into DA Identity from AS and MSCs

**Figure 6:**
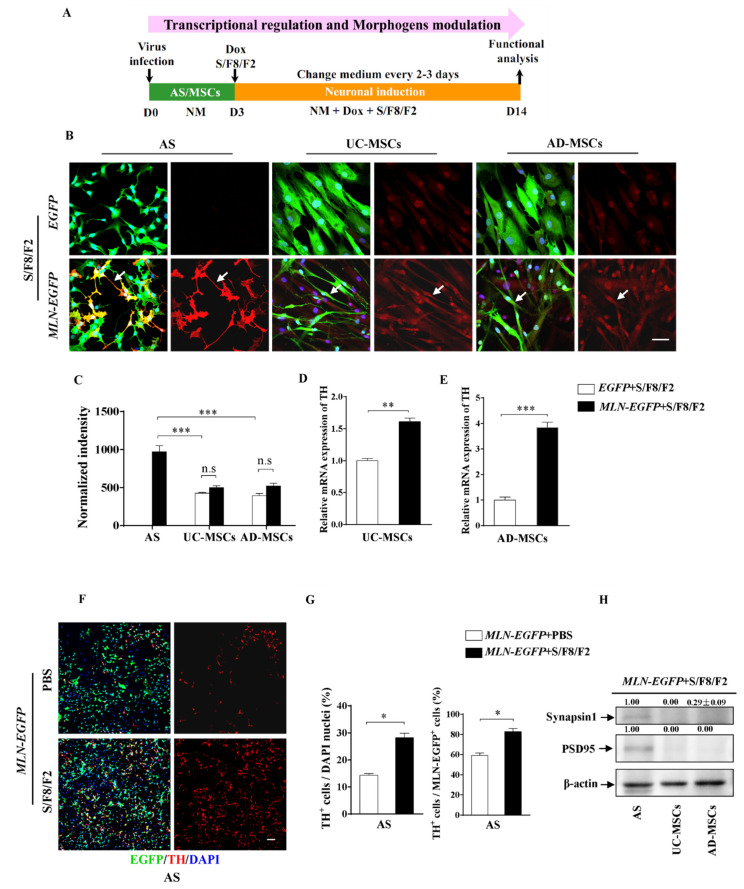
AS exhibited higher TH expression levels as compared to MSCs by the transcriptional and morphogenic activation. (**A**) The paradigm of DA induction by the transcriptional regulation and the morphogenic activation. AS, UC-MSCs, or AD-MSCs were infected with lentivirus carrying *MLN-EGFP* or *EGFP* (control) and then treated with NM containing Dox and morphogens S/F8/F2 to initiate both the transcriptional and morphogenic induction for 14 days. On the 14th day, cells were fixed and stained for double staining of TH and EGFP, and the representative confocal images were shown in (**B**,**F**) (B, AS; UC-MSCs; AD-MSCs). DAPI was used as counterstaining. Note that TH-positive cells with more typical neuronal morphology (indicated with white arrows) could be observed in the AS, UC-MSCs and AD-MSCs treated simultaneously with *MLN* cocktail and S/F8/F2 (The lower panels in B). (**C**–**E**) Quantified data showed the expression level of TH in AS was significantly higher as compared to UC-MSCs and AD-MSCs after the transcriptional and morphogenic induction for 14 days, in addition, the mRNA expression level of TH in UC-MSCs (**D**) and AD-MSCs (**E**) treated simultaneously with *MLN* cocktail, and S/F8/F2 was higher than the control groups with *EGFP* and S/F8/F2 (*n* = 5 independent experiments). (**F**) The representative images at low magnification showed that more TH-positive cells were observed in the AS group with both transcriptional and morphogenic induction for 14 days. (**G**) The effects of morphogens S/F8/F2 treatment on the percentages of TH-positive cells in the whole AS induction system or the *MLN-EGFP*-infected AS at day 14 (*n* = 3 independent experiments). (**H**) Immunoblotting assay for the synaptic makers synapsin1 (75 kDa) and PSD95 (100 kDa) in *MLN-EGFP*+S/F8/F2-treated AS, UC-MSCs, and AD-MSCs after 14-day induction. Note that the expression levels of Synapsin1 and PSD95 in AS were higher than the ones in UC-MSCs and AD-MSCs (*n* = 3 independent experiments). Scale bars: 50 µm in (**B**), 100 µm in (**F**). Data represented as mean ± SEM. * *p* < 0.05, ** *p* < 0.01, *** *p* < 0.001.

It was demonstrated that the regional specification derived from morphogen aims to activate cascades of specific transcriptional networks [43]. Next, we evaluated comprehensively that the role of transcription factors and morphogens in the induction of AS and MSCs into DA identity. The induction procedure was carried out according to the schematic paradigm as in Figure 6A. With the combined modulation of *MLN* cocktail and S/F8/F2, AS produced large numbers of TH-positive neurons with prominent neurites at day 14 (Figure 6B); moreover, significantly more (*p* < 0.05) of TH-positive cells could be observed in the AS culture treated with *MLN* and S/F8/F2 as compared to the one treated with *MLN* alone (Figure 6F,G). These results indicated that morphogens S/F8/F2 promoted the DA neuronal conversion of AS. In line with the previous observation in Figure 4B, AS treated with S/F8/F2 did not develop into any cells with DA neuron phenotype (Figure 6B), confirming a dominant role of *MLN* cocktail in the conversion of AS into DA neurons.

Differently, S/F8/F2 treatment in both UC-MSCs and AD-MSCs culture produced TH-positive cells. The introduction of *MLN-EGFP* not only significantly increased the expression of TH in UC-MSCs (Figure 6D, *p* < 0.01) and AD-MSCs (Figure 6E, *p* < 0.001) but also promoted the morphologic transformation of TH-positive cells into that of primitive neuronal shape with a monopolar-like structure (Figure 6B). These results further supported the view that the differential molecular codes were involved in the induction of AS and MSCs into DA neurons. It is worth noting that the expression level of TH in AS was significantly higher as compared to the levels in UC-MSCs (*p* < 0.001) and AD-MSCs (*p* < 0.001) after 14 days of treatment with both *MLN* cocktail and S/F8/F2. (Figure 6C). Meanwhile, immunoassays against two of the synaptic proteins, synapsin1 and PSD95, showed that the synaptic proteins were expressed in the induced AS but not in the induced UC-MSCs and AD-MSCs (Figure 6H). These data indicated that AS-derived DA neurons, as compared to MSCs-derived TH-expressing cells, displayed more synaptic formation, which is closely related to the maturity of neurons.

## 3. Discussion

**Figure 7:**
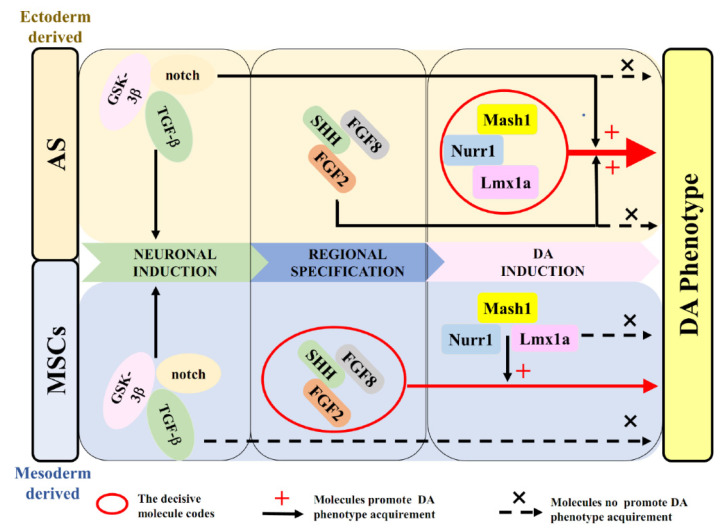
The differential decisive molecular code governing the DA phenotype acquirement of AS vs. MSCs. The diagram summarizes the sequential stages and possible key molecules involved in DA phenotype development from ectoderm- (AS) and mesoderm-derived (MSC) cells, respectively. Transcriptional regulation with Mash1, Nurr1, Lmx1a (MLN) plays a dominant role in the determination of DA phenotype from AS; small molecules involved in TGF-β, GSK-3β, and Notch pathways (SB431542, CHIR99021, and DAPT; S/C/D), as well as morphogens SHH, FGF2 and FGF8 (S/F8/F2), accelerate the efficiency of DA induction. In contrast, morphogens S/F8/F2 involved in DA regional specification are the key molecules for the DA phenotype acquirement from MSCs; Transcriptional cocktail MLN could promote the quality of DA phenotype derived from MSCs. Possible interactions between this network of molecules are indicated by arrows with solid lines. Molecules that do not promote DA phenotype acquirement are indicated by arrows with dashed lines. The decisive molecular codes are indicated by red circles and arrows with solid lines.

In the last few decades, mechanisms involving the developmental process of DA neurons have been elaborately described. The establishment of DA neurons is orchestrated by intrinsic transcriptional factors and environmental cues with a number of stages: neuronal induction, regional patterning/specification, and DA induction [23,30,43,45,47,48]. Chemicals S/C/D [19], morphogens S/F8/F2 [24,25,49] and transcription-factor cocktail MLN [16,17,19,33], which have been reported in a series of studies, promoted the sequential developmental stages of DA generation (Figure 7). To our knowledge, this study is the first of its kind, where we have directly compared the molecular codes between AS and MSCs induction into DA neurons. Unexpectedly, we found that different key molecules determined the induction process from these two kinds of cells. As summarized in Figure 7, transcriptional regulation with MLN plays a dominant role in the determination of conversion from AS into DA neurons, whereas morphogens S/F8/F2 determine whether MSCs express DA phenotypes by induction. Besides the different determinant factors, all the molecules involving the establishment of DA neurons contributed to the induction process to some extent (Figure 7). From the perspective of evolution, AS and MSCs are derived from ectoderm and mesoderm, respectively, which possibly explains why the different key molecules are required for the DA phenotype determination. Note that the induction from MSCs to DA neurons crossed germinal layers, which indicated the morphogens (S/F8/F2) involved in the regional patterning/specification would be critical for the trans-germinal layer cell fate determination [50].

Many studies have demonstrated that transplantation of MSCs could facilitate motor improvement in an animal of PD [12,51,52]; however, it remains unclear to what extent the induction of MSCs into DA neurons is involved in the alleviation of PD symptoms. The paracrine effects of MSCs seem to play a critical role in the therapeutic effects of MSCs since the recovery of DA neurons could also be observed by transplantation with naive MSCs [12,52,53,54]. In the present study, although we found that S/F8/F2 induced MSCs to DA neuron-like cells, it was worth noting that the induced cells remained the original morphology with moderate TH expression levels. A similar expression pattern was found in many other reports on MSC-induced DA neurons [11,24,49,55]. Even in the optimized in vitro conditions, the induced MSCs developed monopolar neuron-like cells without complex neurites, lacking the synaptic structure of the functional neurons. It is deducible that the MSCs-induced DA neuron-like cells probably do not fulfill all the characteristics of the functional DA neurons. These observations were consistent with the reports by Pires et al. and McCoy et al., which reinforced the view that paracrine effects rather than cell substitution are involved in the therapeutic effects of MSCs in PD models [1,46,56].

In comparison with mesodermal-derived MSCs, AS belong to the ectodermal cell type. AS distribute extensively in the brain, providing abundant cell resources for the conversion strategy. Especially in PD pathology, the progressive degeneration of DA neurons is accompanied by the occurrence of AS reactive hyperplasia [57]. Our data indicated that AS acted as differentiated somatic cells, displayed tremendous superiorities in the lineage-specific induction into DA neurons as compared with pluripotent MSCs. AS-converted DA neurons showed elaborate morphology of neurons with high levels of TH expression. With the addition of chemicals S/C/D or morphogens S/F8/F2, the conversion rates of AS to DA neurons in vitro exceeded approximately 80% in the MLN expressing AS at day 14. Especially, AS-derived DA neurons, under the transcriptional and morphogenic induction for 14 days, expressed the synaptic makers synapsin1 and PSD95. These data suggest that AS, after the careful manipulation of the intrinsic and extrinsic cues, holds great potential as a candidate for DA neurons substitution therapy.

Indeed, a series of reports have revealed the feasibility of AS-to-neuron strategy in cell therapy for neurodegenerative disease. Cervo et al. has reported the successful induction of functional DA neurons in vitro and in vivo through the combined regulation of MLN and miRNA218 [19]. Xiang et al. has reported the direct AS-to-neuron conversion in the cortex by the introduction of the neuronal differentiation 1 (NeuroD1) transcription factor [58]. The same team has also reported an in vivo conversion technology reprogramming striatal AS into GABAergic neurons through ectopic expression of NeuroD1 and distal-less homeobox 2 (Dlx2) transcription factors [59]. Qian et al. reported an in situ converted nigra neuron strategy from AS by depleting the RNA-binding protein polypyrimidine tract binding protein 1 (PTBP1) showed alleviation of PD symptoms [60]. However, despite all these positive reports, the in vivo cell fate conversion strategy remained disputed. Very recently, a report by Zhang et al. described that the lineage-traced AS were not converted into neurons in vivo by either introduction of *NeuroD1* or knockdown of *PTBP1* [61]. As we clearly showed in the present study, the enriched AS in vitro mainly expressed astrocytic markers GFAP and S-100β and could be efficiently converted into DA neurons by both the intrinsic and extrinsic regulations. The observation, together with the reports on in vivo conversion, suggest that the AS-to-neuron strategy in vitro is feasible, but the complex microenvironment cues in vivo and the delivery way of exogenous genes probably has to be further elucidated for better in vivo conversion effects.

As for cell-based therapies for PD, better DA candidates for grafting have always been pursued. DA differentiation from iPSCs and ESCs is another important strategy for PD cell therapy [62]. A number of protocols were developed in which iPSCs/ESCs were induced into DA lineage by the chemical modulation or transcriptional regulation. Most of the efficient protocols for DA induction used a combination of several pharmacological compounds [6,25,63]. However, the varying induction efficiency, ranging from 4% to 95% of the final cell numbers, has been reported by the different studies [64,65]. In comparison, the transcriptional induction of iPSCs/ESCs seemed to generate more of a homogeneous DA population from multiple iPSC lines [66] or from mouse/human ESCs [67]. Lmx1a, which was proved in the present study to be critical for AS conversion into DA neurons, plays a dominant role in the DA differentiation of iPSCs/ESCs [66,67]. The iPSCs/ESCs-derived DA cultures exhibited morphological and molecular properties of mature DA neurons, showing similarity to AS-derived DA neurons. Nevertheless, the tumorigenicity by incomplete differentiation, immune response and ethical issues are still obstacles of the clinical application of ESC/iPSCs strategy in PD therapy [62,68]. AS, originated from the somatic cell source, show superiority of safety over the iPSCs/ESCs. In any case, a further attempt for stable therapeutic effects and safety has to be carried out before any of these cell candidates was introduced into the clinical application of PD cell therapy.

## 4. Materials and Methods

### 4.1. Plasmid Construction and Viral Production

*Tet-O-FUW-Mash1-P2A-Lmx1a-F2A-Nurr1-IRES-EGFP* (*MLN-EGFP*) plasmid and *Tet-O-FUW--IRES-EGFP* (*EGFP*) plasmid were kind gifts from Dr. Nilima Prakash (Helmholtz Zentrum Munich). The *Ngn2* fragment of cds sequence was synthesized according to the Genebank sequence NM_024019 (by GENEWIZ Biotechnology company, Suzhou, China) and further cloned to produce *Tet-O-FUW-Ngn2-IRES-EGFP* (*Ngn2*) plasmid.

The *EGFP*, *MLN-EGFP*, *Ngn2-EGFP*, *FUW-rtTA2* (Addgene, 20342) lentiviruses were generated in HEK 293T cells co-transfected with the three package plasmids: *pVSV-G* (Addgene, 12259), *pMDLg* (Addgene, 12251), and *pRSV-Rev* (Addgene, 12253) using polyethylenimine (PEI) (Polysciences, Warrington, FL, USA; 24765-1). Viral particles were concentrated by ultracentrifugation in a Thermo LYNX-6000 centrifuge with A27-8 × 50 rotor at 50,000× *g* for 120 min at 4 °C. The pellet was gently resuspended in PBS, aliquoted, and stored at −80 °C.

### 4.2. The Primary Culture of AS, AD-MSCs and UC-MSCs

The primary astrocytic culture was prepared from postnatal 3 to 5 days Sprague–Dawley rats (SPF Biotechnology Co, Beijing, China). Hippocampi were separated on ice, followed by incubation with 0.05% trypsin (Sigma, Saint Louis, MO, USA; T3924) for 10 min at 37 °C. The cells were seeded in DMEM (Gibco, Waltham, MA, USA; C11995500BT) medium containing 10% FBS (Gibco, Waltham, MA, USA; 10099-141) and 1% penicillin/streptomycin (P/S). After reaching 90%–100% confluency, the primary culture was shaken at 225 rpm/min at 37 °C for 12 h and washed with cold PBS [41]. After discarding the supernatant, the residual cells were trypsinized and replated in astrocytic growth medium containing DMEM, 10% FBS, 1% P/S, and 5 ng/mL FGF2 (STEMCELL Technologies, Vancouver, BC, Canada; 78003.2). Experiments were performed on AS during their 3rd to 5th passage in vitro.

All studies involved with human samples were performed in accordance with the ‘Ethical Guiding Principles on Human Embryonic Stem Cell Research’ (of the Ministry of Science and Technology and the Ministry of Health, China, 2003) and Helsinki Declaration. Umbilical cords and adipose tissues were generously donated with the signed informed consent and ethical approval from the first affiliated hospital of Soochow University. UC-MSCs were isolated and cultured as previously described by Zhang et al. [69]. Briefly, the umbilical cord vessels and outer membrane were removed, and mesenchymal tissue in Wharton’s jelly was dissected and minced into 0.5 cm^3^ pieces. The small pieces were inoculated in 10 cm diameter culture dishes covered with DMEM/F12 medium (Gibco, Waltham, MA, USA; C11330500BT) containing 10% FBS and 1% P/S. For AD-MSCs [52], the adipose tissue was washed, cut into small pieces after removing the blood vessels and connective tissue under an anatomical microscope. The blocks were digested with 0.3 pzu/mL type I collagenase (Sigma, Saint Louis, MO, USA; C0130) at 37 °C for 30 min, followed by centrifugation at 500× *g* for 10 min. The cell pellet was suspended with DMEM with 10% FBS and 1% P/S and seeded at the density of 8 × 10^4^/cm^2^. Experiments were performed on UC-MSCs and AD-MSCs during the 3rd to 8th passage in vitro.

The assay for the osteogenic and adipogenic differentiation capacity of MSCs was performed as described previously [69].

### 4.3. Flow Cytometry Analysis

A total of 1 × 10^5^ MSCs were harvested and incubated with either PE, FITC, APC/cy7, or PerCP conjugated antibodies against CD105, CD45, CD90, and CD34 mouse anti-human monoclonal antibodies and appropriate isotype controls with human MSCs analysis kit (Biolegend, San Diego, CA, USA). Stained cells were analyzed using a flow cytometer (BD LSRFortessa, Franklin Lakes, NJ, USA), and data were analyzed using FlowJo software version 10.0 (BD Biosciences, Franklin Lakes, NJ, USA).

### 4.4. Immunofluorescence

Cultured cells were fixed 4% paraformaldehyde, permeabilized with 0.15% Triton X-100 in PBS (PBST). blocked with 3% FBS in PBST, followed by incubation with the following primary antibodies overnight at 4 °C: anti-GFP (Abcam, Cambs, UK; ab13970; 1:1000), anti-Tuj1 (Abcam, Cambs, UK; ab78078; 1:1000), anti-TH (Abcam, Cambs, UK; ab112; 1:1000 or Santa Cruz, Heidelberg, Germany; sc-25269; 1:100), anti-GFAP (Abcam, Cambs, UK; ab7260; 1:1000), anti-S-100β (Sigma, Saint Louis, MO, USA; S2532; 1:250), anti-Nestin (Abcam, Cambs, UK; ab134017; 1:1000), anti-PAX6 (Abcam, Cambs, UK; ab195045; 1:500), anti-MAP2 (Sigma, Saint Louis, MO, USA; M4403; 1:1000 or Abcam, Cambs, UK; ab32454, 1:200). Immunoreactivity was visualized using appropriate Alexa Fluor-conjugated secondary antibodies and observed using the confocal microscope (Nikon, Tokyo, Japan, A1R HD25). The cell nuclei were visualized by staining with DAPI (Beyotime, Shanghai, China; C1002) at 0.5 μg/mL for 10 min.

### 4.5. Cell Quantification

Cells were analyzed with Image Pro Plus software version 6.0 (Media Cybernetics, Bethesda, MD, USA). Cell counts were performed on 9 individual microscopic fields (0.072 mm^2^), randomly chosen across 2 diameters of each coverslip (×400 magnification). An average of 1000 cells or 100 transfected cells was sampled on each coverslip; results shown represent values from 6 to 9 coverslips/treatment from 3 independent experiments.

### 4.6. Western Blot

Cells were lysed in the cell lysis buffer (Beyotime, Shanghai, China; P0013). After determination of protein concentration (Lowry method), samples were separated by SDS-PAGE on 10%–15% polyacrylamide gels and transferred to nitrocellulose membranes. Membranes were blocked in PBS containing 5% non-fat milk and 0.2% Tween-20, and incubated overnight with the following primary antibodies: anti-TuJ1 (Abcam, Cambs, UK; ab78078; 1:1000), anti-TH (Abcam, Cambs, UK; ab112; 1:1000), anti-MAP2 (Sigma, Saint Louis, MO, USA; M4403; 1:1000), anti-synapsin1 (Sysy, Gottingen, Germany; 106011; 1:500), anti-PSD95 (Sysy, Gottingen, Gemany; 124003; 1:200) and anti-Actin (Santa Cruz, Heidelberg, Germany; sc-8432; 1:1000), specific protein bands were visualized by enhanced chemiluminescence (GE Life Sciences, Freiburg, Germany), after incubation with appropriate horseradish peroxidase-IgG conjugates (Bioss, Woburn, MA, USA; bs-0296D-HRP; 1:5000). The bands were quantified with Image Pro Plus software version 6.0 (Media Cybernetics, Bethesda, MD, USA) after subtraction of the local background.

### 4.7. RT-PCR

RNA was extracted with Total RNA Kit I (Omega Bio-tek, Norcross, GA, USA; R6834-01) and reverse-transcribed into cDNA using Prime Script RT reagent kit (TAKARA Bio, Kusatsu, Japan; RR036A). Quantitative PCR was performed on Bio-Rad CFX96 Real-Time PCR System by using TB Green Premix Ex Taq (TAKARA Bio, Kusatsu, Japan; RR042B) and 2^−ΔΔCT^ method. The primers were described as follows: TH-F: CGGATGAGGAAATTGAGAAGCTG and TH-R: AGACAGGCAGTGCAGGAGCTC; β-actin was used as an internal control.

### 4.8. Transcriptional Factors-Based Neuron Induction

The cells were infected with lentiviruses (AS: MOI = 2; UC-MSCs or AD-MSCs: MOI = 5) in the Opti-MEM medium (Gibco, Waltham, MA, USA; 11058021) supplemented with 4 µg/mL of polybrene (YESEN, Shanghai, China; 40804ES76) for 5–6 h. Cells were then transferred to the neuronal induction medium (NM) containing neurobasal medium (Gibco, Waltham, MA, USA; 10888022), 1% N2 (Gibco, Waltham, MA, USA; 7156) 2% B27 (Gibco, Waltham, MA, USA; 17504044), 1% Gluta-MAX (Gibco, Waltham, MA, USA; 35050061), 10 ng/mL BDNF (Peprotech, East Windsor, NJ, USA; AF-450-02), 10 ng/mL GDNF (Peprotech, East Windsor, NJ, USA; AF-450-51), and 50 µM ascorbic acid (Peprotech, East Windsor, NJ, USA; 5088177). On the 3rd day after infection, doxycycline (Dox; 2 µg/mL, YESEN, Shanghai, China, 60204ES08) was added to the NM to activate the expression of the exogenous genes. The NM was changed every 2–3 days till the 14th day (D14) for functional analysis (Figure 2A).

### 4.9. Chemicals and Morphogens Induction

Cells were primed in the NM for 3 days before the treatment with chemical cocktails S/C/D or morphogens S/F8/F2. The dosage are as follows: 5 µM SB431542 (Peprotech, East Windsor, NJ, USA; 3014193), 1.5 µM CHIR99021 (Peprotech, East Windsor, NJ, USA; 2520691), 5 µM DAPT (Peprotech, East Windsor, NJ, USA; 2088055), 250 ng/mL SHH (Peprotech, East Windsor, NJ, USA; 100-45-25), 100 ng/mL FGF8 (Peprotech, East Windsor, NJ, USA; 100-25A-25), and 50 ng/mL FGF2 (Peprotech, East Windsor, NJ, USA; 100-18B-10). The NM was changed every 2–3 days, and the fresh chemicals S/C/D or morphogens S/F8/F2 were added to the NM, respectively (Figure 3A and Figure 4A).

### 4.10. Statistical Analysis

Numerical data were presented as mean ± SEM. The data were subjected to 2-tailed Student t-tests or analysis of variance and the appropriate post-hoc analysis using GraphPad Prism version 6.0 (GraphPad Software Inc., San Diego, CA, USA). The level of significance was preset at *p* < 0.05.

## Data Availability

The data that support the findings of this study are available from the corresponding author upon reasonable request.
